# Supplementary material for: Facile Hydrothermal Synthesis of SnO2 Nanoflowers for Low-Concentration Formaldehyde Detection
Source: Nanomaterials (Basel). 2022 Jun 21;12(13):2133. doi: 10.3390/nano12132133 (PMC9267946; doi:10.3390/nano12132133)
Supplement: Supplementary file 1 [file nanomaterials-12-02133-s001.zip › nanomaterials-1763811 - supplementary.pdf]

nanomaterials

MDPI

Supplementary Materials

Facile Hydrothermal Synthesis of SnO_2 Nanoflowers for Low-Concentration Formaldehyde Detection

Chao Xiang [1,†], Tingting Chen [2,3,†], Yan Zhao [1], Jianhai Sun [2,*], Kaisheng Jiang [2,3], Yongzhen Li [4], Xiaofeng Zhu [4], Xinxiao Zhang [2,3], Ning Zhang [1] and Ruihua Guo [4,*]

[1] State Key Laboratory of Nuclear Power Safety Monitoring Technology and Equipment, China Nuclear Power Engineering Co., Ltd., Shenzhen 518172, China; xiangchao@cgnpc.com.cn (C.X.); zhaoy@cgnpc.com.cn (Y.Z.); ningzhang@cgnpc.com.cn (N.Z.)
[2] State Key Laboratory of Transducer Technology, Aerospace Information Research Institute, Chinese Academy of Sciences, Beijing 100194, China; chentingting19@mails.ucas.ac.cn (T.C.); jiangkaisheng20@mails.ucas.ac.cn (K.J.); zhangxinxiao19@mails.ucas.ac.cn (X.Z.)
[3] School of Electronic, Electrical and Communication Engineering, University of Chinese Academy of Sciences, Beijing 100049, China
[4] Institute of Urban Safety and Environmental Science, Beijing Academy of Science and Technology, Beijing 100054, China; liyongzhen@bmilp.com (Y.L.); zxf_402@163.com (X.Z.)
* Correspondence: sunjh@aircas.ac.cn (J.S.); guoruihua@bmilp.com (R.G.)
† These authors contributed equally to this work.

Citation: Xiang, C.; Chen, T.; Zhao, Y.; Sun, J.; Jiang, K.; Li, Y.; Zhu, X.; Zhang, X.; Zhang, N.; Guo, R. Facile Hydrothermal Synthesis of SnO_2 Nanoflowers for Low-Concentration Formaldehyde Detection. *Nanomaterials* **2022**, *12*, 2133. https://doi.org/10.3390/nano12132133

Academic Editor: Abdelhamid Elaissari

Received: 25 May 2022
Accepted: 16 June 2022
Published: 21 June 2022

Publisher's Note: MDPI stays neutral with regard to jurisdictional claims in published maps and institutional affiliations.

Copyright: © 2022 by the authors. Licensee MDPI, Basel, Switzerland. This article is an open access article distributed under the terms and conditions of the Creative Commons Attribution (CC BY) license (https://creativecommons.org/licenses/by/4.0/).

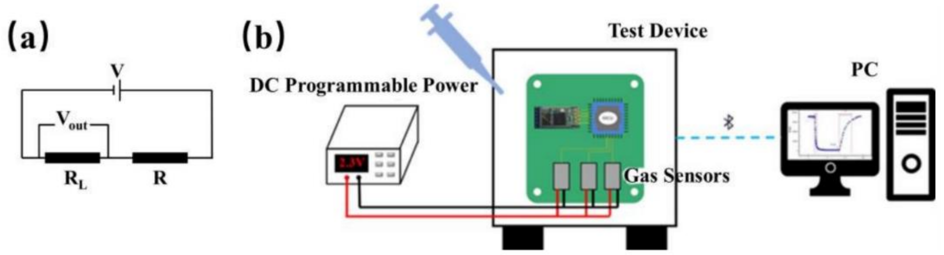

Figure S1. Schematic of the fabrication process for MEMS HCHO sensors. (**a**) Measuring circuit of MEMS sensors, (**b**) the test system of the MESM gas sensor.

Measurement of the Gas Sensor Resistance

The gas sensing properties of the fabricated gas sensors for HCHO were measured by recording the electrical resistance variation of the gas-sensitive element and calculated according to the definition. According to the electric circuit of the gas sensor shown in Figure S1a, the resistance of the MEMS sensor can be calculated via the Equation: $R = (V - V_{out}) R_L/V_{out}$, where V is the circuit voltage, which was set at 5 V during the test, V_{out} is the output voltage of the gas-sensing analysis system, and RL is the load resistor.

The Test System of the MEMS Gas Sensor

The sensing method is that by applying a certain heating voltage on the fabricated MEMS electrodes, a desired temperature on the chip could be obtained. The input DC voltage of the MHP was provided by DC Programmable Power (IT6432, ITECH), and the real-time output signal of the MHP was measured with LabVIEW on the computer through Bluetooth, as shown in Figure S1b.

Calculation Method for Different Concentrations of Test Gases

In our work, the purity of gases was calculated according to the stationary-state gas distribution method. the desired concentrations of HCHO (C) were obtained by diluting gas standard material of HCHO (100 ppm) with air as background gas calculated as $C = V_s \times C_s/V$, where V_s was the volume of standard gas that was injected into the chamber, C_s was

the concentration of standard gas (100 ppm standard gas mixing with clean air, V was the volume of the sealed chamber (1 L). The humidity of the test environment was 33% RH
